# Sociality does not drive the evolution of large brains in eusocial African mole-rats

**DOI:** 10.1038/s41598-018-26062-8

**Published:** 2018-06-15

**Authors:** Kristina Kverková, Tereza Bělíková, Seweryn Olkowicz, Zuzana Pavelková, M. Justin O’Riain, Radim Šumbera, Hynek Burda, Nigel C. Bennett, Pavel Němec

**Affiliations:** 1 Department of Zoology, Faculty of Science, Charles University, Viničná 7, CZ-128 44 Praha 2, Czech Republic; 2 Department of Biological Sciences, University of Cape Town, 7701 Rondebosch, South Africa; 3 Department of Zoology, Faculty of Science, University of South Bohemia, Branišovská 1760, CZ-370 05 České Budějovice, Czech Republic; 4 Department of General Zoology, Faculty for Biology, University of Duisburg-Essen, 45117 Essen, Germany; 5 Mammal Research Institute, Department of Zoology and Entomology, University of Pretoria, Pretoria, 0002 South Africa

## Abstract

The social brain hypothesis (SBH) posits that the demands imposed on individuals by living in cohesive social groups exert a selection pressure favouring the evolution of large brains and complex cognitive abilities. Using volumetry and the isotropic fractionator to determine the size of and numbers of neurons in specific brain regions, here we test this hypothesis in African mole-rats (Bathyergidae). These subterranean rodents exhibit a broad spectrum of social complexity, ranging from strictly solitary through to eusocial cooperative breeders, but feature similar ecologies and life history traits. We found no positive association between sociality and neuroanatomical correlates of information-processing capacity. Solitary species are larger, tend to have greater absolute brain size and have more neurons in the forebrain than social species. The neocortex ratio and neuronal counts correlate negatively with social group size. These results are clearly inconsistent with the SBH and show that the challenges coupled with sociality in this group of rodents do not require brain enlargement or fundamental reorganization. These findings suggest that group living or pair bonding *per se* does not select strongly for brain enlargement unless coupled with Machiavellian interactions affecting individual fitness.

## Introduction

The social brain hypothesis (SBH) contends that the demands imposed on individuals by living in cohesive social groups exert a selection pressure favouring the evolution of large brains and complex cognitive abilities^1^. It was originally proposed to explain the exceptional cognitive abilities in primates, but it has since been extended to a wider range of vertebrate taxa, including cetaceans, carnivores, bats, insectivores, ungulates, various birds and cichlids (for a review see^2,3^). While the SBH has gained great traction in evolutionary anthropology, what the underlying mechanisms are, or how broadly it applies to other animals remains an area of active research. Recent studies incorporating phylogenetic corrections and more stringent measures have failed to provide strong support^4–7^ and even new analyses in primates, incorporating a substantially larger number of species and phylogenetic uncertainty, challenge its validity^8,9^. An exception is a recent study reporting larger brain size in cetaceans living in mid-sized groups^10^. The hypothesis has only recently been tested in rodents for the first time and the results revealed that, in ground squirrels, sociality is not associated with larger relative brain size, but that social species tend to have larger bodies and correspondingly absolutely larger brains^6^, suggesting that a possible link between body size and sociality may be mediating the effect on brain size.

Over the past decades, different factors have been proposed as the main driving force of cognitive enhancement mediated by sociality in birds and mammals generally, and primates in particular (reviewed in^2,11^). The original idea emphasized competition and tactical deception (as reflected in the name “Machiavellian intelligence”)^12^, but the mechanism was later reformulated by Dunbar and Shultz^13,14^ as the need to maintain group cohesion through individual recognition and affiliative interactions to diffuse conflict. According to this latter view, cognitively demanding social behaviours are believed to take the form of behavioural coordination and pair bond formation in non-primates, but might become generalized to all group members in primates (reviewed in^2^). Mating system thus represents another domain of sociality that is pertinent to brain evolution. Indeed, association between monogamy and larger relative brain size has been reported in ungulates, carnivores, and birds^13,15^. Cooperative breeding itself is another factor that has been suggested as potentially facilitating large brain evolution^15–17^ (but see^18,19^).

Despite recent progress in comparative methods that take phylogenetic relatedness into account, broad comparative studies, while allowing for greater statistical power, remain inherently prone to spurious findings due to large variations in ecology and life history traits, the unrecognized influence of hidden variables, heterogeneity in evolutionary trajectories and selection pressures, and data inconsistencies across datasets^3,9,20,21^. One way to limit the effects of biological heterogeneity and statistical interference is to study brain evolution within closely related but behaviourally diverse clades^21^. Here, we use this approach and test the SBH in African mole-rats (Rodentia: Bathyergidae). This group is ideal to provide insights into some of the unanswered questions without introducing confounding factors associated with differences in general biology and ecology that have been implicated in brain size evolution. Major factors besides sociality include substrate use, habitat complexity, diet and foraging mode, activity pattern, home range, developmental mode and maternal investment (for a review, see^20^). Mole rats are uniform in most of these traits. They are all strictly subterranean, burrowing and feeding on underground parts of plants^22–26^, but cover the whole social spectrum, from strictly solitary to the remarkably social cooperative breeders, warranting the term “eusocial”^27,28^. They all give birth to altricial young and from the limited information available, it seems there are no systematic differences in maternal investment (gestation length, litter size, lactation length) connected to sociality^29^. The naked mole-rat is somewhat exceptional, though, in having substantially larger litters than the other species^30^. Solitary species, however, seem to be seasonal breeders^31–33^, in contrast to mostly aseasonally breeding social species^34–36^. Sociality also goes hand in hand with larger burrow systems and thus increased “home range”, but reliable data for all species are not available and there is substantial intraspecific variation^37,38^.

Solitary mole-rats are highly territorial and aggressive towards conspecifics. Their affiliative social interactions are confined to short periods of time during the breeding season and maternal care for juveniles, which disperse shortly after weaning^31–33^. Social species live in stable, multigenerational families in which only few individuals (often just a single bonded pair) reproduce and most of their offspring stay permanently within the family as non-reproductive helpers. Typically, members of this cohesive group cooperate through digging and maintaining the burrows, foraging for food and bringing it to communal storage, engaging in colony defence against intruders and predators, and taking care of the pups – grooming, huddling, returning them to the nest chamber when they wander off and providing them with cecotrophs^22,39–43^. In the genus *Cryptomys* the groups tend to be smaller and much less stable, especially in the mesic parts of the range^44^. Moreover, social mole-rats, in contrast to solitary ones, seem to be monogamous^45–48^, which is another purported driver of cognitive abilities in non-primate mammals^13^. There is also evidence of individual recognition^43,49^ and elaborate vocalization and social interactions in the social species^30,50–52^ so these are not just simple aggregations. Mole-rat sociality is based on long-term (lifelong in eusocial species) pair bonds and stable social relationships among all members of an extended family^27,28,53^. Due to limited opportunity for dispersal and new burrow formation, there seems to be little flux in the composition of the social group, especially in eusocial species, colonies of which are characterized by extensive overlap of adult generations and permanent (lifelong) philopatry^27^. Importantly, manipulative or Machiavellian behaviour is likely selected against in mole-rat colonies with monopolized reproduction because it would harm an individual’s inclusive fitness.

While social environment is a complex system, where various components come into play, some patterns in the data could provide insight into their relative importance. The general prediction is that monogamous social species of mole-rats should have bigger brains than solitary species. If social bonding, individual recognition, maintaining group cohesion and cooperation exert the major selection pressure^13,54^, then the eusocial species with extremely high reproductive skew towards a single breeding pair might be expected to show the largest brains and cognitive potential, since they live in the largest and most cohesive groups, with a decreasing trend towards the solitary end of the social spectrum. If, however, the competitive aspect of sociality is more important, eusocial species should not face a pressure to increase brain size, since outcompeting other colony members would not improve an individual’s fitness. Mole-rats that are still social, but not with such an extreme reproductive skew (genus *Cryptomys*)^44,55^, could perhaps be expected to show greater cognitive capacities and larger brains, since they could potentially benefit by becoming dominant and taking over or starting their own colony, or realise their direct fitness by extra-colonial paternity^55^. However, as noted above, it is highly unlikely that complex Machiavellian interactions are present in mole-rats. No difference in brain size between the groups would thus indirectly point to these competitive interactions being the most important factor.

The social organization of eusocial mole-rats resembles that of eusocial insect societies in several aspects, such as monopolization of reproduction^27,28^ and division of labour among non-reproductive group members^39,56–58^ (but see^59,60^). Alternative hypotheses for social brain evolution have been recently developed for (eu)social insects^61^ and African mole-rats have been suggested as a possible vertebrate group where they may apply. The distributed cognition hypothesis (DCH) seems to be particularly pertinent, as its predictions are opposite to those of the SBH. It assumes that in multi-generational colony groups characterized by high reproductive skew and therefore subjected to strong colony-level selection, members can rely on social communication to supplement individual cognition. The hypothesis therefore predicts relaxed selection for individual cognitive abilities and reduced brain investment in such (eu)social species^61^. If cooperative information sharing among individual mole-rats outweighs within-colony conflicts, solitary species should have the largest brains, with a decreasing trend toward the eusocial end of the social spectrum, where the potential for “distributed cognition” is highest.

Most comparative studies dealing with the SBH published to date have focused on relative brain mass or volumes of specific brain regions (particularly the neocortex) and the results were largely based on the analysis of previously published data^5,13,54,62–65^. In this study, we test predictions of the SBH and the DCH, using new, unprecedentedly comprehensive data on brains of 11 species representing all six existing genera of mole-rats. In light of recent studies on cognition^66,67^ and neuronal scaling rules^68,69^, it becomes clear that regarding cognitive abilities as a function of relative brain size is a gross oversimplification, and might be even misleading^70^. There are at least two factors at play – brain size and neuronal density^69,71^. Thus, at neuroanatomical level, more cognitive power can be achieved by increasing brain size or size of specific brain regions, or by increasing the neuronal density without that necessarily manifesting as a substantial increase in volume. Investigating a broad range of brain size measures enables us to pinpoint which brain parts, if any, are under selection, or if the whole brain responds in concert.

## Results

### Absolute and relative brain size

While it might be possible that subterranean microphthalmic mammals are somehow aberrant in the way their brains are built, we show that this is not a concern in the choice of mole-rats as our model group. With the exception of the naked-mole rat, bathyergids do not significantly differ from other rodents in either their allometric brain-body relationship or previously published neuronal scaling rules (Fig. S1). Notably, the naked mole-rat not only has a smaller brain than expected for a rodent of its body size, but also a lower number of neurons than predicted for its brain size.

**Figure 1:**
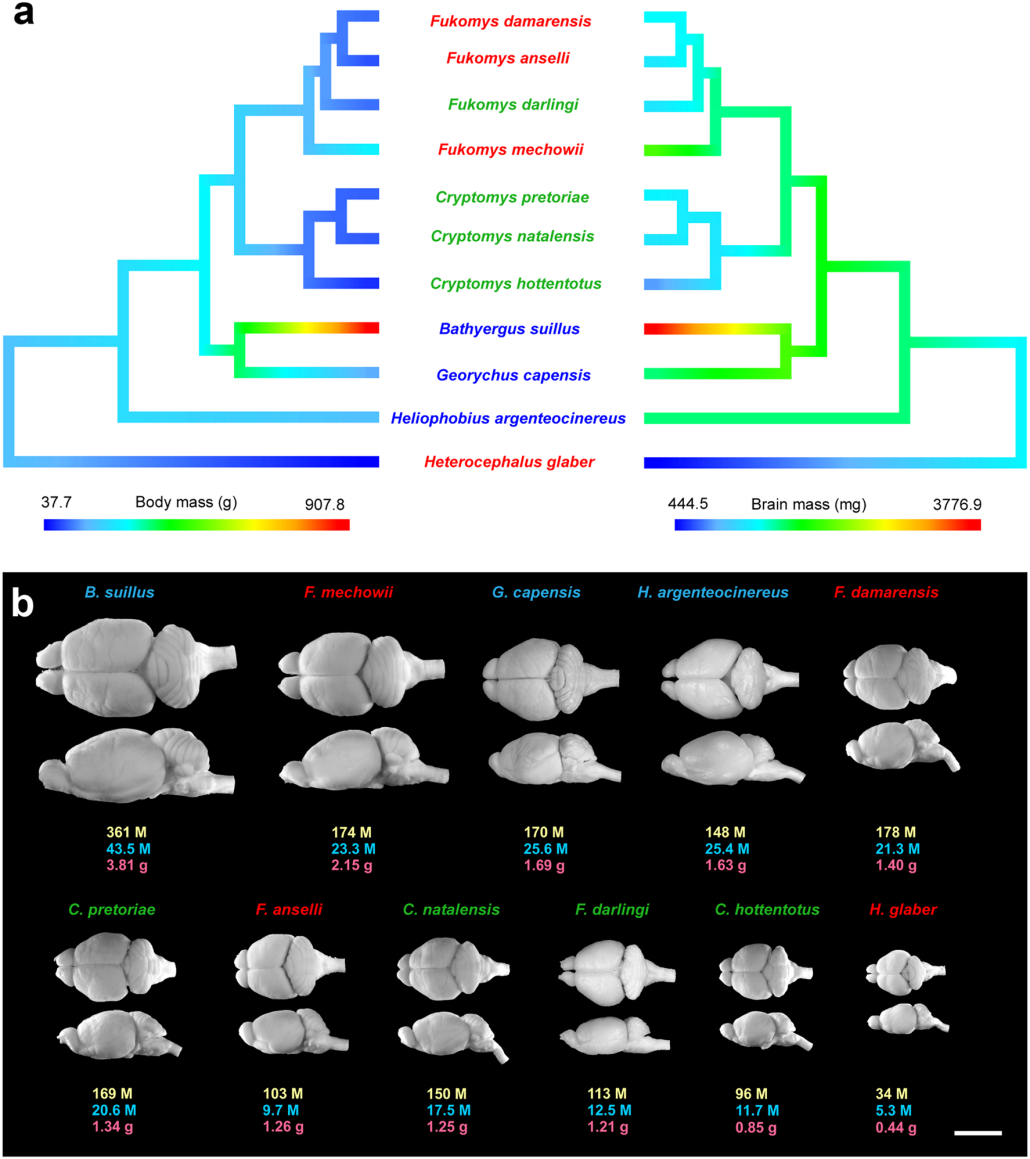
Body size, brain size and number of neurons for the mole-rat species examined. (**a**) The phylogeny of the 11 African mole-rat species included in the analyses with body mass (the left tree) and brain mass (the right tree) mapped as a continuous trait with the ancestral states reconstructed using the phytools package in R. The topology of the tree follows a published report^113^. (**b**) Dorsal and lateral views of representative brains are accompanied by information concerning total numbers of brain neurons (yellow), numbers of pallial neurons (blue) and brain mass (red). M, million. Scale bar, 10 mm. Species names are colour-coded by sociality: red – eusocial, green – social, blue – solitary.

**Figure 2:**
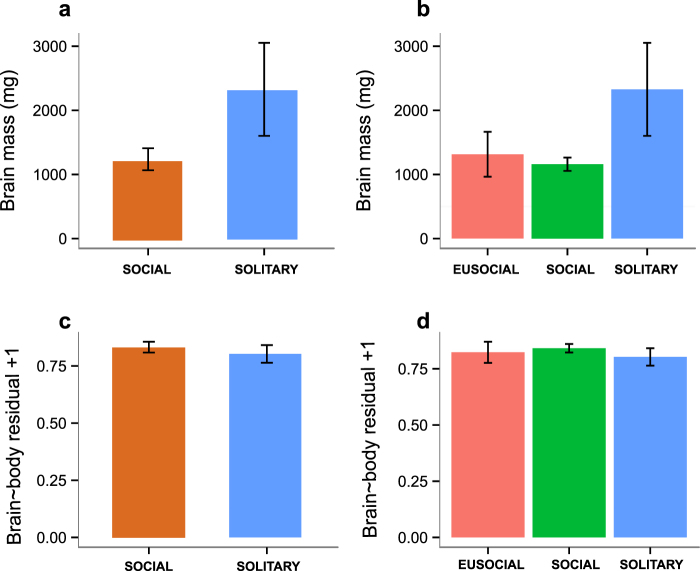
Absolute and relative brain size by sociality. Bar plots illustrating the differences in absolute (**a**,**b**) and relative brain size (**c**,**d**) between social and solitary (left column graphs) and eusocial, social and solitary species of African mole-rats (right column graphs). Note that solitary mole-rats tend to have absolutely, but not relatively larger brains than social ones. Relative brain size is expressed as a residual from the brain-on-body regression line for Rodentia, with 1 added to get positive numbers. Data are represented as mean ± SEM.

The studied species range in average body mass from 38 g to 908 g and in average brain mass from 0.44 g to 3.81 g (Fig. 1, Table S1). Solitary species have significantly larger body mass than social species (posterior mean = 1.1089, CI = [0.1481, 2.2049], pMCMC = 0.0321, lambda mean = 0.75; for other comparisons, see Table S2). Likewise, absolute brain mass tends to be higher in solitary species, although the difference is not significant (posterior mean = 0.6486, CI = [−0.0018, 1.4556], pMCMC = 0.0741, lambda mean = 0.84; for other comparisons, see Table S2) (Fig. 2a,b).

Relative brain size, a measure previously shown to be associated with sociality^2,13,72^ (expressed as a residual from the regression line for rodents) shows no connection to the social system in mole-rats (Fig. 2c,d; for statistics, see Table S2).

### Volumetric analyses

**Figure 3:**
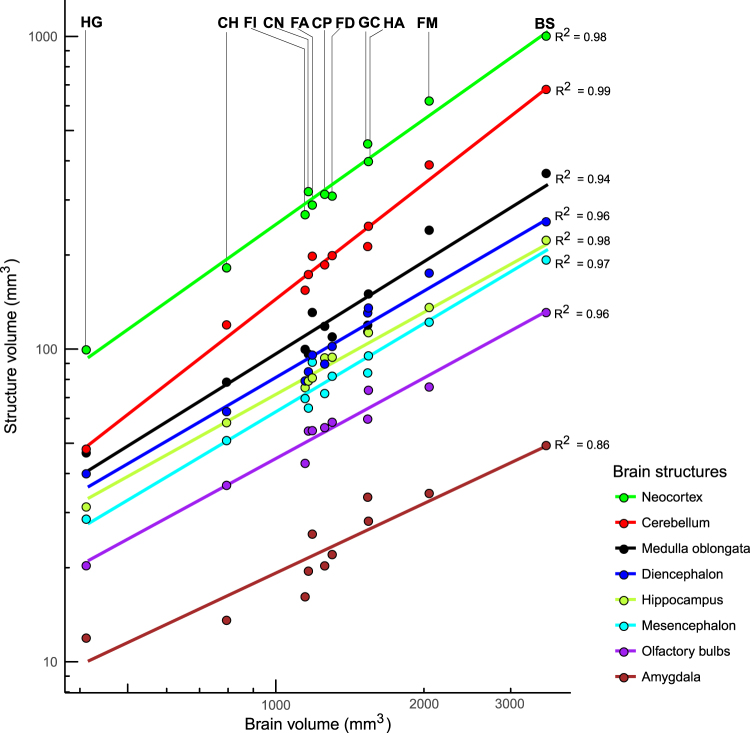
Scaling of selected brain structures with brain volume. Log-transformed structure volumes are plotted against log-transformed total brain volumes. The diencephalon volume was calculated as the sum of the thalamic and hypothalamic volumes, the mesencephalon volume as the sum of the tectal and tegmental volumes. Fitted lines and coefficients of determination are taken from the OLS regressions of species averages. Note that all structures scale very predictably with total brain volume. BS, *Bathyergus suillus*; CH, *Cryptomys hottentotus*; CN, *Cryptomys natalensis*; CP, *Cryptomys pretoriae*; FA, *Fukomys anselli*; FD, *Fukomys damarensis*; *FI*, *Fukomys darlingi*; FM, *Fukomys mechowii*; GC, *Georychus capensis*; HA, *Heliophobius argenteocinereus*; HG, *Heterocephalus glaber*.

To assess whether there is any evidence of mosaic evolution (disproportional enlargement of specific brain parts, see e.g.^73^) in response to selective pressures associated with sociality, we measured the volumes of 14 brain regions and determined the scaling rules for those structures with brain size (Tables S1 and S3). All measured volumes correlate significantly and very tightly with whole brain volume (Fig. 3). In fact, brain volume accounts for over 90% of variance in all structure volumes measured, except for the amygdala (*R*^2^ = 0.86) (Table S3). We then compared relative volumes of these brain structures between sociality grades. Not surprisingly, given the high proportion of variance explained by brain size, relative volumes of all the structures are independent of sociality (Table S4). Mole-rats are thus no exception to the broad rule that conserved scaling rules explain an overwhelming proportion of variance in brain region volumes, as has been clearly shown in a much larger sample of mammals^74^.

**Figure 4:**
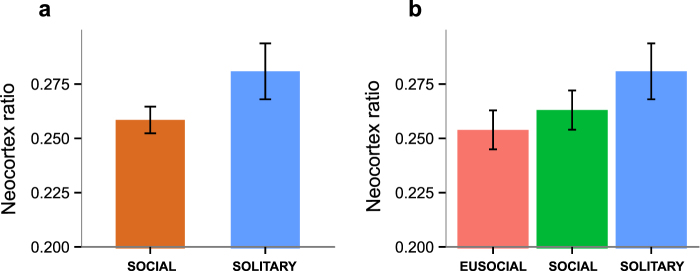
Neocortex ratio by sociality. Bar plots illustrating the differences in neocortex ratio (the ratio of neocortex volume to the rest of the brain volume) between (**a**) social and solitary, and (**b**) eusocial, social and solitary species of African mole-rats. Data are represented as mean ± SEM. Note that solitary species tend to have higher neocortex ratios than social ones.

**Figure 5:**
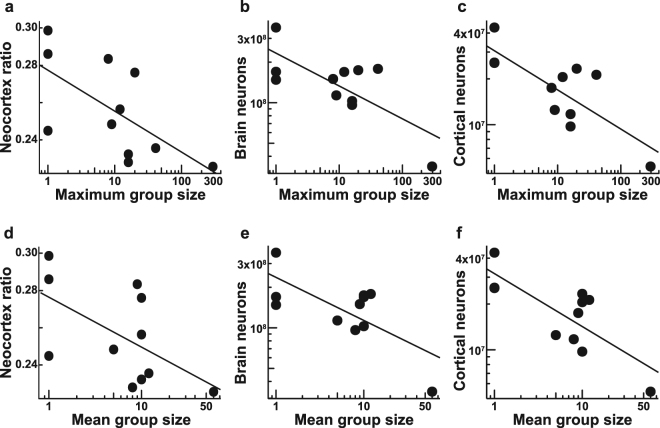
The relationship of selected neuronal correlates of cognitive capacity and social group size. Scatter plots showing negative correlation between neocortex ratio (**a**,**d**), number of brain neurons (**b**,**e**), number of cortical neurons (**c**,**f**) and maximum (**a**–**c**) and mean group size (**d**–**f**). The fitted lines represent the phylogenetic least squares regressions.

The neocortex ratio [C_R_: neocortex volume/(brain volume − neocortex volume)] has been traditionally used as a proxy for intelligence in tests of the SBH. We found that in mole-rats, there are no significant differences between the social categories, but there is a potential trend towards higher C_R_ in solitary species (Fig. 4, Table S2). C_R_ also decreases significantly with maximum group size (PGLS: −0.0278, p = 0.0294; Fig. 5a) and mean group size (PGLS: −0.0358, p = 0.0218; Fig. 5d), but the relationship is not significant after removing the naked mole-rat from the analysis (maximum group size: −0.0297, p = 0.0721; mean group size: −0.0337, p = 0.1405).

### Number of neurons

**Figure 6:**
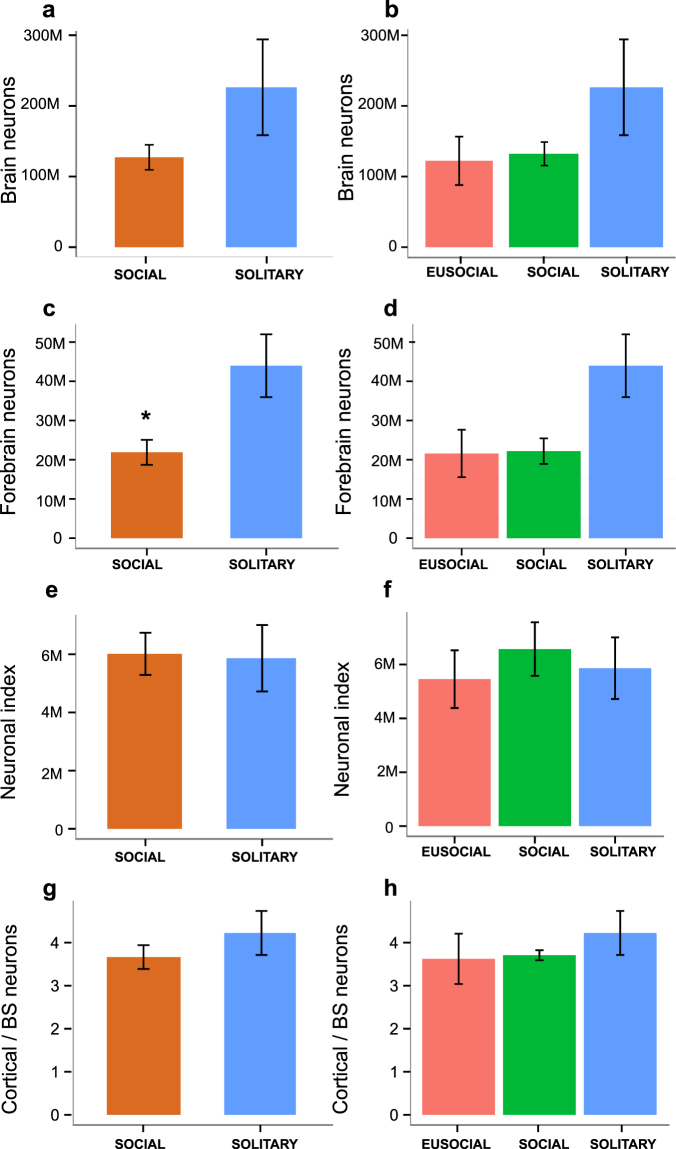
Neuronal approximations of cognitive capacity by sociality. Bar plots illustrating the differences in the average number of brain neurons (**a**,**b**), the average number of forebrain neurons (**c**,**d**), neuronal index (**e**,**f**) and the ratio of cortical neurons to brain stem neurons (**g**,**h**) between social and solitary (left column graphs) and between eusocial, social and solitary species of African mole-rats (right column graphs). Note that solitary mole-rats have significantly more forebrain neurons and tend to have more brain neurons and higher cortical neurons ratios than social ones. The neuronal index is expressed as a residual from the neurons-on-body mass regression line for Rodentia, adjusted by adding the largest negative value to get positive numbers. Data are represented as mean ± SEM; asterisk marks a significant difference (95% confidence interval does not include 0).

Neuronal numbers in the whole brain and specific brain regions are presented in Fig. 1 and Table S5, results of the statistical analyses in Table S6. Mole-rats generally conform to the neuronal scaling rules previously established for rodents^75^ (Fig. S1b,c). Solitary species tend to have higher absolute numbers of neurons compared to social species (Table S6; Fig. 6a,b). Importantly, this difference is most pronounced and statistically significant in the number of cortical neurons (posterior mean = 0.7928, CI = [0.0694, 1.5191], pMCMC = 0.0396, lambda mean = 0.48) and neurons in the subcortical forebrain (posterior mean = 0.6884, CI = [0.0306, 1.3882], pMCMC = 0.0480, lambda mean = 0.44), i.e., solitary species have significantly more neurons in the forebrain (posterior mean = 0.7603, CI = [0.0405,1.4421], pMCMC = 0.0332, lambda mean = 0.48) (Fig. 6c,d).

Consistent with these results, the number of brain neurons decreases with both maximum group size (PGLS: −0.2167, p = 0.0322; Fig. 5b) and mean group size (PGLS: −0.2804, p = 0.0492; Fig. 5e), although this relationship is not significant after removing the naked mole-rat from the analysis (maximum group size: −0.1423, p = 0.1048; mean group size: −0.1643, p = 0.133). Number of cortical neurons also decreases with maximum group size (PGLS: −0.2724, p = 0.0019; Fig. 5c) and mean group size (PGLS: −0.3680, p = 0.0021; Fig. 5f), and, notably, this relationship remains significant even when analysed without the naked mole-rat (maximum group size: −0.2905, p = 0.0272; mean group size: −0.2342, p = 0.018).

**Figure 7:**
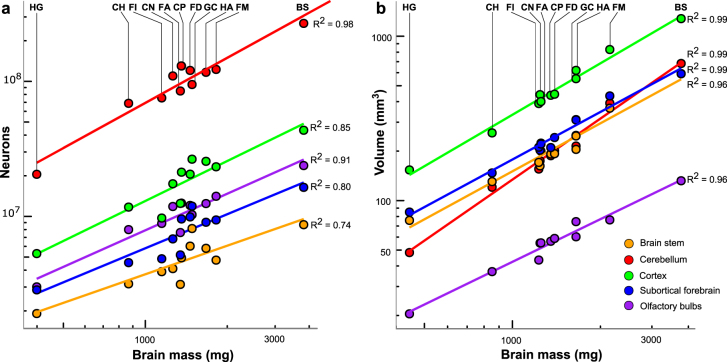
Scaling of neuronal numbers and volumes of major brain divisions with brain mass. (**a**) Number of neurons contained in the brain divisions plotted as a function of brain mass. (**b**) Division volumes plotted as a function of brain mass, for comparison. Data points correspond to species averages. Coefficients of determination are reported for the OLS regressions. See caption to Fig. 3 for abbreviations.

Numbers of neurons contained in the brain regions examined correlate significantly and very tightly with their mass (Table S3) and, because the size of these regions scales highly predictably with brain size (Fig. 7b), also with brain mass (Fig. 7a). Numbers of neurons relative to the brain mass do not differ between the social grades in the whole brain or any of the five brain parts (Table S6).

We also examined residuals from the neurons-body regression line for rodents, essentially the neuronal index proposed by Herculano-Houzel^76^ as an adequate proxy for cognitive abilities, and the ratio of cortical neurons to the neurons in brain stem, another index of cognitive power, analogous to the neocortex ratio (Fig. 6e–h). No significant differences were found between the solitary and social groups for either the neuronal index (posterior mean = −0.2467, CI = [−1.7914, 1.2497], pMCMC = 0.72, lambda mean = 0.07; for other comparisons, see Table S2) or cortical neurons ratio (posterior mean = 0.4113, CI = [−0.0363, 0.8235], pMCMC = 0.0585, lambda mean = 0.36; for other comparisons, see Table S2), although there is a trend for higher cortical neurons ratio in solitary species (Fig. 6g,h).

## Discussion

The analyses performed in this study do not indicate a positive association between the neuroanatomical correlates of brain information processing capacity and sociality in African mole-rats. Despite examining measures ranging from overall brain size to neuronal numbers, we found no differences between the social grades in any of the relative measures, whether previously reported (relative brain size, neocortex ratio)^13,62^, or tested for the first time (neuronal index, cortical neurons ratio). The few significant differences we revealed relate to absolute measures and were in favour of solitary mole-rats. Most importantly, solitary species have more neurons in the forebrain than social ones. Because the forebrain subserves higher cognitive functions and because the number of forebrain neurons is one of the major determinants of brain computational capacity^69,71,77^, the high number of forebrain neurons likely endows solitary species with improved cognitive abilities and increased behavioural flexibility. General cognitive abilities aside, it could be hypothesized that social mole-rats would have relatively larger brain areas related to individual recognition and/or emotional processing, such as olfactory areas or the amygdala^78,79^. This is not the case, however. Brain structure scaling is very conservative in mole-rats and we found no evidence of mosaic evolution. These results show that social living that entails maintaining group cohesion, individual recognition, behavioural coordination, monogamous pair bonding and cooperative breeding does not drive the evolution of large brains harbouring large numbers of neurons in African mole-rats. Importantly, our failure to find support for the SBH is not due to lack of statistical power. If that were the case, there would be no significant results and the trends would be in the opposite direction.

Although the debate about the importance of relative vs. absolute brain size for cognition is still ongoing and recent evidence for both is available^66,80^, our results do not support the SBH in any case. Since we included both absolute and relative measures of whole brains and several brain regions, the results are not tied to any particular assumptions about the neural substrate for cognitive capacity. Drawing an analogy with insect eusociality (see Introduction), it is tempting to interpret the lower number of forebrain neurons in social mole-rats as evidence supporting the DCH. The very fact that the naked mole rat, the species that forms the largest colonies of up to 295 members^24^ and in which non-breeding individuals of both sexes are physiologically suppressed from reproduction^81,82^, has the smallest brain and the lowest number of neurons (both in absolute and relative terms; Tables S1 and S5, Fig. S1) is in line with the hypothesis. However, in contrast to DCH predictions^61^, a reduced brain size and lower numbers of neurons were not observed in the other eusocial species, in which reproductive skew is maintained solely by incest avoidance^43^ or by combination of incest avoidance and a suppression of female reproductive physiology^83^. While it is well possible that physiological reproductive suppression of non-breeders is necessary to achieve the level of group selection needed to relax the selection for individual cognitive abilities, alternative explanations cannot be excluded. For instance, the small, hairless and semi-poikilotermic^84^ naked mole-rat may face more severe metabolic constraints than its larger hairy relatives. All other differences between social and solitary species reported in this study seem to be attributable to differences in body size. Taken together, the results obtained in this study are inconsistent with the SBH and do not provide a sound support for the DCH, they highlight the importance of viewing body size not just as a confounding factor to be corrected for, but as intrinsically connected with and driving brain size and computational capacity. Technically, body size is tightly coupled to absolute brain size and that, in turn, with the total number of neurons. There is substantial evidence and growing consensus that the total number of neurons and their densities are decisive for brain computational power^67,69,71,77^. Moreover, it has been posited that increased numbers of neurons lead to increased brain complexity, as neurons are the brain’s “computational units” and more neuronal assemblies can be created, a notion supported by recent experimental evidence in mice^85^.

The special case of mole-rats might also provide an insight into a more general problem with the SBH. Considering that, across vertebrates, the single best determinant of brain size is body size^86^, we might have to deal with a confounding factor responsible for driving both sociality and larger bodies. Because the evolution of group-living is generally believed to have evolved as a response to predation^3,87^, which can select for greater body size^88,89^, and a growing body of evidence suggests that predation also directly selects for larger brains, it has been suggested by van der Bijl and Kolm (2016) that predation may confound the SBH by causing spurious correlation between sociality and brain size^3^. The subterranean niche confers relative protection from predators and predation is not a driver of social evolution in mole-rats (see below). Therefore, we argue that low predation pressure in subterranean burrows may partly explain the lack of positive relationship between the correlates of brain processing capacity and sociality in African mole-rats.

These findings add to the series of recent papers that have reported no link between relative brain size and sociality in mammals^5,6,8,9,80^ (but see^10^) and fish^4,90^. However, they are in stark contrast to previous studies in primates, cetaceans, carnivores and insectivores^62–65,91^ that have found a positive relationship between C_R_ and social group size. In mole-rats, the trend goes in the opposite direction: solitary species tend to have larger C_R_ and C_R_ tends to correlate negatively with group size. This makes sense in light of the findings of Schillaci^92^, who reports that C_R_ in primates correlates highly positively with body size and is not a significant predictor of group size, after controlling for body size. In other words, C_R_ is in fact indicative of absolute brain size, and that is what drives the correlation in primates. Interestingly, a recent test of the SBH in another rodent group (ground squirrels of the tribe Marmotini)^6^, revealed that there is no link between relative brain size and sociality, but that social species tend to be larger and hence have absolutely larger brains. This relationship between body mass and sociality (and, correspondingly, the neocortex ratio) is opposite in mole-rats, and thus contrary to the SBH. Once again, these results point to a tight coupling between body size and absolute brain size. The latter seems to be generally linked with the brain’s intrinsic complexity: the proportional and absolute size of the neocortex, the number of cortical areas and the total number of cortical neurons increase with absolute brain size (for reviews, see^93,94^).

The results presented here in no way challenge the existence of more subtle neurobiological differences between solitary and social mole-rats. Indeed, differences in neuropeptide receptor distributions and densities and in adult hippocampal neurogenesis were reported^95–97^, though only limited data on a handful of species are currently available. Likewise, our findings cannot rule out that sociality does select for larger brains in mole-rats, as all we can observe is the end result of all selective pressures and constraints put together. Some hidden factors might be confounding the results, since not enough reliable data is available on all aspects of life-history in mole-rats. However, from the information available, there does not seem to be a systematic difference in maternal investment (gestation length, litter size, weaning age) between social and solitary species^29^. Solitary species, however, are seasonal breeders, in contrast to mostly aseasonally breeding social species^31–36^. To our knowledge, this has not been previously linked to differences in brain size, but it is another difference that cannot be separated from sociality and deserves further investigation.

Furthermore, it is possible that solitary mole-rats are subject to selection for larger size, or that social mole-rats face some constraints on body and/or brain size that the solitary ones are free from. Factors contributing to mole-rat sociality, or lack thereof, are still not well understood, although the aridity food distribution hypothesis is currently the prevalent explanation^53^ (for alternative explanations, see^27^). Social mole-rats, generally living in harsher environments with fewer resources, may be prevented from attaining larger body (and brain) size due to the need to reduce energetic demands. Brains are metabolically expensive^98^ and, simultaneously, excavating the burrow systems, especially in hard soils, carries an enormous energetic cost^99^. Lowering the metabolic demands might therefore be of utmost importance. Smaller body size and communal foraging means improving the chances of subsisting on scarce and dispersed food sources. The fact that this reduction in body size is not accompanied by an increase of relative brain size (which would result purely from decelerated brain mass reduction compared to body mass reduction) suggests that sociality does not exert enough selective pressure on brain size to outweigh these metabolic constraints. This is not to say that sociality does not act on cognitive abilities, but its importance may be more limited than generally assumed by the SBH.

To conclude, the absence of any evidence for selection acting on larger brain size or higher neuronal numbers in eusocial mole-rats, the pinnacle of cooperative breeding in vertebrates, weakens the notion that behavioural coordination or stable bonding is cognitively demanding and drives the evolution of cognitive capacity across vertebrates^13^. The fact that the challenges coupled with sociality do not entail brain enlargement or fundamental reorganization in this group resonate with an alternative view that dyadic and polyadic social interactions might not require flexible cognitive solutions in real-time, but could be solved by simpler evolved rules-of-thumb^100^. To our knowledge, there is no evidence that mole-rats engage in any Machiavellian interactions. But even if they were involved in sophisticated strategies like formation of coalitions or tactical deception, such behaviours would not increase individual fitness in species with monopolized reproduction; hence Machiavellian interactions should not effectively select for larger brains and improved cognitive abilities in eusocial mole-rats. Taken together, mole-rat sociality involves most putative drivers of cognitive abilities except for Machiavellian interactions. Therefore, our findings suggest, albeit indirectly, that Machiavellian interactions rather than social bonding and cooperation underlie the previously found link between social complexity and brain size.

Future stringent tests assessing the validity and generality of the SBH should encompass both (i) broad-scale comparative analyses incorporating various measures of social complexity as well as ecological and life-history variables including potentially confounding factors (such as appropriate proxies of predation pressure) and (ii) studies of variation in brain composition among closely related species that have similar ecologies and life-history traits but exhibit different levels of sociality. It will be equally important to direct further efforts to move from using readily measured traits such as brain size to more reliable proxies for cognitive abilities such as neuronal numbers and sizes of brain regions involved in specific behaviours. Integration of these approaches will provide deeper insights into the causal relationship between brain processing capacity and sociality.

## Methods

### Animals

African mole-rats (Bathyergidae) are endemic to sub-Saharan Africa. They form a monophyletic group within the rodent clade Ctenohystrica. Recently, it was suggested that the naked mole-rat *Heterocephalus glaber* be moved into its own family Heterocephalidae based on the time of divergence and distinctive morphological and genetic traits^101^. Since this taxonomical revision does not change the phylogenetic relationships in any way, all the species are treated here as belonging to the monophyletic family Bathyergidae.

Eleven species of African mole-rats were examined: the Cape dune mole-rat *Bathyergus suillus* (BS), silvery mole-rat *Heliophobius argenteocinereus* (HA), Cape mole-rat *Georychus capensis* (GC), common mole-rat *Cryptomys hottentotus* (CH), Natal mole-rat *Cryptomys natalensis* (CN), highveld mole-rat *Cryptomys pretoriae* (CP), Ansell’s mole-rat *Fukomys anselli* (FA), Mashona mole-rat *Fukomys darlingi* (FI), Damaraland mole-rat *Fukomys damarensis* (FD), giant mole-rat *Fukomys mechowii* (FM) and naked mole-rat *Heterocephalus glaber* (HG). All animals were adults and in the case of cooperatively breeding species non-reproductive individuals. Reproductive animals are usually the largest in the colony and can even substantially increase their body size after gaining reproductive status^102–105^. On the other hand, it is highly unlikely that reproductive status has any significant effect on absolute brain size or composition, because all reproductive animals are recruited from helpers well after reaching maturity. As reproductive individuals were not available in sufficient numbers and for all species, they were excluded from the analysis because including them could introduce a potential bias in relative brain size. Both sexes were close to equally represented (females: 52, males: 49, unknown: 4), with at least one male and one female of each species for each analysis. Animals were obtained either from colonies in the University of Duisburg-Essen, the University of South Bohemia (České Budějovice) and the University of Cape Town or wild-caught and housed at the University of Pretoria and University of Cape Town. Details on origin and use of experimental animals are provided in Table 1.

**Table 1:** Origin and use of the experimental animals.

Species	Origin of Animals				Experimental Use		
	Colony			Wild-caught	Volumetry	Isotropic fractionator	Brain-body relationship
	Duisburg-Essen	České Budějovice	Cape Town				
*Heterocephalus glaber*	7	3	3		5	3	13
*Bathyergus suillus*				11	5	3	11
*Georychus capensis*				8	4	3	8
*Heliophobius argent*.		8			4	3	8
*Fukomys mechowi*	8	3			3	3	11
*Fukomys anselli*	9				3	3	9
*Fukomys damarensis*				11	4	3	11
*Fukomys darlingi*		3		4	4	3	7
*Cryptomys hottentotus*				8	4	3	8
*Cryptomys pretoriae*				10	5	3	10
*Cryptomys natalensis*				13	5	3	13
Total	24	17	3	65	46	33	106

Animals were killed by halothane overdose and perfused transcardially with heparinized phosphate-buffered saline, followed by 4% phosphate-buffered paraformaldehyde (PFA). Brains were dissected and weighed immediately after perfusion, post-fixed overnight in the same fixative, and stored in 0.5% PFA or in anti-freeze solution at −20 °C until further processing.

All experimental procedures were conducted in accordance with the Guidelines for Animal Care and Treatment of the European Union, and were approved by the animal care and ethics representatives of the Faculty of Science of Charles University in Prague, University of Duisburg-Essen and University of Pretoria (AUCC 030110-002, AUCC 040702-015 and AUCC 000418-006). Captive animals originated from breeding colonies, the maintenance of which was approved by the Veterinary Office of the City of Essen, Germany (AZ: 32-2-1180-71/328) and by Ministry of Agriculture of the Czech Republic (22395/2014-MZE 17214); wild animals were collected under permit from the relevant Nature Conservation authorities of Gauteng, Western Cape and Northern Cape Provinces, South Africa. All efforts were made to minimize animal numbers and suffering.

### Sociality

Given the lack of a generally accepted measure of social complexity and problems associated with even simple measures such as group size^106^, we decided to adopt a simple approach and treat sociality either as a binary variable (solitary: BS, GC, HA; social: all others), or a categorical variable with tree levels (solitary: BS, GC, HA; social: CH, CN, CP, FI; eusocial: FA, FD, FM, HG). While crude, it is not subject to intraspecific variation and research effort bias and the categories also roughly correspond to group size^24^. The categories were delimited based on reproductive skew (the number of overlapping generations). Although it remains controversial whether solitary or social life-style is ancestral for African mole-rats^27^, eusociality has evolved at least two times, once in the naked mole-rat and once within the genus *Fukomys* (Fig. 1a). Social group size (see electronic supplementary material, Table S7) was also used in a subset of analyses for the sake of comparison with earlier studies.

### Relative brain size

A total of 106 animals were used to investigate the brain-body scaling in African mole-rats. The interspecific allometry of brain mass was determined by ordinary least square (OLS) linear regression of brain mass on body mass. Brain-body allometry at the order level (Rodentia) was used to calculate residuals for mole-rats. This relationship was based on a separate dataset of brain and body masses for rodent species (n = 414) collated from the literature (for references, see Dataset S1). The regression line is thus kept independent of the data and provides an unbiased reference.

### Volumetric analysis

Forty-five brains were used to perform the volumetric analysis. Brains were embedded in gelatine blocks fixed in sucrose-paraformaldehyde solution (30% sucrose, 4% PFA) and sectioned on a cryostat in the coronal plane at a thickness of 60 µm. Every second section was mounted on a slide and stained with cresyl violet. Total brain volume and the volume of 14 distinct regions of the brain (olfactory bulbs, olfactory cortices, neocortex, entorhinal cortex, hippocampus, amygdala, striatum, septum, thalamus, hypothalamus, midbrain tectum, midbrain tegmentum, cerebellum and medulla oblongata) were determined. Contours of the brain and the measured regions were drawn from the sections using a camera lucida. These drawings were then digitized using a Wacom tablet and the areas measured using the Scion Image software. The total area of the drawn structures was multiplied by the section thickness and sampling ratio to obtain the structure volume. Final volumes were then corrected for shrinkage. The extent to which a brain shrinks during histological processing is different in each brain. To obtain comparable values, each structural volume was multiplied by a correction factor (C_ind_) calculated for each brain as follows: C_ind_ = volume of the perfused brain/sum of serial section volumes. The volume of the perfused brain was calculated by dividing the brain mass by the fixed brain tissue density (1.036 g/cm^3^)^107^. Note that brain mass does not change significantly within the first hours of fixation^107^. Because all brains used in this study were weighed immediately after perfusion, i.e., after very short fixation, these measurements correspond to mass/volume of fresh brain.

### Isotropic fractionator

Three brains per each species (33 in total) were used for quantification of total numbers of cells, neurons and nonneuronal cells using the isotropic fractionator method^108^. Brains were postfixed in 4% PFA for at least two weeks. After fixation, brains were dissected into the following five compartments: olfactory bulbs, cerebral cortex (including the underlying white matter and comprising the neocortex, hippocampus, olfactory cortices such as piriform and entorhinal cortex, and pallial amygdala), subcortical forebrain (comprising the diencephalon, caudate putamen, nucleus accumbens, globus pallidus, ventral pallidum, olfactory tubercle and septum), cerebellum, and brain stem (comprising the mesencephalon and medulla oblongata). Each dissected brain division was homogenized in 40 mM sodium citrate with 1% Triton X-100 using Tenbroeck tissue grinders (Wheaton, Millville, NY, USA). When turned into an isotropic suspension of isolated cell nuclei, homogenates were stained with the fluorescent DNA marker DAPI, adjusted to a defined volume, and kept homogenous by agitation. The total number of nuclei in suspension, and therefore the total number of cells in the original tissue, was estimated by determining the density of nuclei in small fractions drawn from the homogenate. At least four 10 µl aliquots were sampled and counted using a Neubauer improved counting chamber (BDH, Dagenham, Essex, UK) with an Olympus BX51 microscope equipped with epifluorescence and appropriate filter settings; additional aliquots were assessed when needed to reach the coefficient of variation among counts ≤0.15. Once the total cell number was known, the proportion of neurons was determined by immunocytochemical detection of the neuronal nuclear marker NeuN^109^. This neuron-specific protein was detected by an anti-NeuN rabbit polyclonal antibody (Merck Millipore, dilution 1:800). The binding sites of the primary antibody were revealed by a secondary anti-rabbit antibody conjugated with Alexa Fluor 594 (Life Technologies, Carlsbad, CA, USA; dilution 1:400). An electronic hematologic counter (Alchem Grupa, Torun, Poland) was used to count simultaneously DAPI-labelled and NeuN-immunopositive nuclei in the Neubauer chamber. A minimum of 500 nuclei was counted to estimate the percentage of double-labelled neuronal nuclei. Numbers of nonneuronal cells were derived by subtraction.

### Data analysis

All data analyses were performed in R Studio with R 3.3.2.^110^. Prior to statistical analyses data were log-transformed. For estimating the differences between social and non-social species (sociality as a fixed effect), we used Bayesian generalised linear mixed models with Markov chain Monte Carlo (MCMC) estimation in the package MCMCglmm^111^, with phylogenetic correction and multiple measurements per species taken into account as random effects. The lambda parameter was estimated for each MCMC model. This parameter potentially varies between 0, indicating that the trait evolution is independent of phylogeny, and 1, indicating that the traits are evolving according to Brownian motion on the given phylogeny, while intermediate values correspond to an effect of phylogeny weaker than under the Brownian model^112^. Mole-rat phylogeny was constructed from a published report^113^. Each model was run for 5 million iterations, with a burnin of 5000, and a thinning interval of 1000, that means approximately 5000 estimations were sampled. Convergence was confirmed by visual inspection of trace plots. Estimates of the differences between the levels of sociality were calculated from a posterior distribution created by subtracting the estimates for each level obtained during each MCMC iteration. Parameter estimates were considered statistically significant when 95% credible intervals (CI) did not include 0.

All linear regression coefficients, used to describe allometric scaling relationships, were determined by the ordinary least squares (OLS) method from species averages. For analyses of the relationship between selected brain measures and social group size, phylogenetic least squares (PGLS) method implemented in the R package nlme^114^ was used with Pagel’s lambda model for scaling the phylogenetic variance-covariance matrix. Statistical significance was evaluated at α level of 0.05.

Relative sizes and indexes of cognitive power were calculated as follows: relative brain size as a residual from the brain-body mass OLS regression for 414 species of rodents, excluding mole-rats; relative volumes of brain regions as residuals from the OLS regression of the brain region volume on the whole brain volume; relative numbers of neurons as residuals from the neurons-brain mass OLS regression for mole-rats; the neuronal index as residuals from the neurons-body mass OLS regression for rodents^76^, excluding mole-rats; the cortical neurons ratio as the ratio of the number of cortical neurons to the number of brain stem neurons.

### Data availability

All data generated or analysed during this study are included in this published article (and its Supplementary Information files).

## Electronic supplementary material

Electronic Supplementary Material

Dataset S1
