# Supplementary material for: Sociality does not drive the evolution of large brains in eusocial African mole-rats
Source: Sci Rep. 2018 Jun 15;8:9203. doi: 10.1038/s41598-018-26062-8 (PMC6003933; doi:10.1038/s41598-018-26062-8)
Supplement: Supplementary file 1 — Electronic Supplementary Material [file 41598_2018_26062_MOESM1_ESM.docx]

**Electronic Supplementary Material**

**Sociality does not drive the evolution of large brains in eusocial African mole‑rats**

**Kristina Kverková, Tereza Bělíková, Seweryn Olkowicz, Zuzana Pavelková, M. Justin O'Riain, Radim Šumbera, Hynek Burda, Nigel C. Bennett, and Pavel Němec**

**Corresponding author:**

**Pavel Němec**

E-mail: pgnemec@natur.cuni.cz

Department of Zoology, Faculty of Science, Charles University in Prague, Viničná 7,

CZ-128 44 Praha 2, Czech Republic

**Content:**

1. **Figure S1.** Brain–body scaling and neuronal scaling rules for mole-rats compared with those for other rodents
2. **Table S1.** Brain mass and brain region volumes
3. **Table S2.** Statistical analyses of body and brain sizes and indexes of cognitive power
4. **Table S3.** Scaling rules for brains of the African mole-rats
5. **Table S4.** Statistical analyses of brain region volumes
6. **Table S5.** Numbers of neurons in the whole brain and the major brain divisions
7. **Table S6.** Statistical analyses of neuronal numbers
8. **Table S7.** Mean and maximum group size for mole-rat species

**
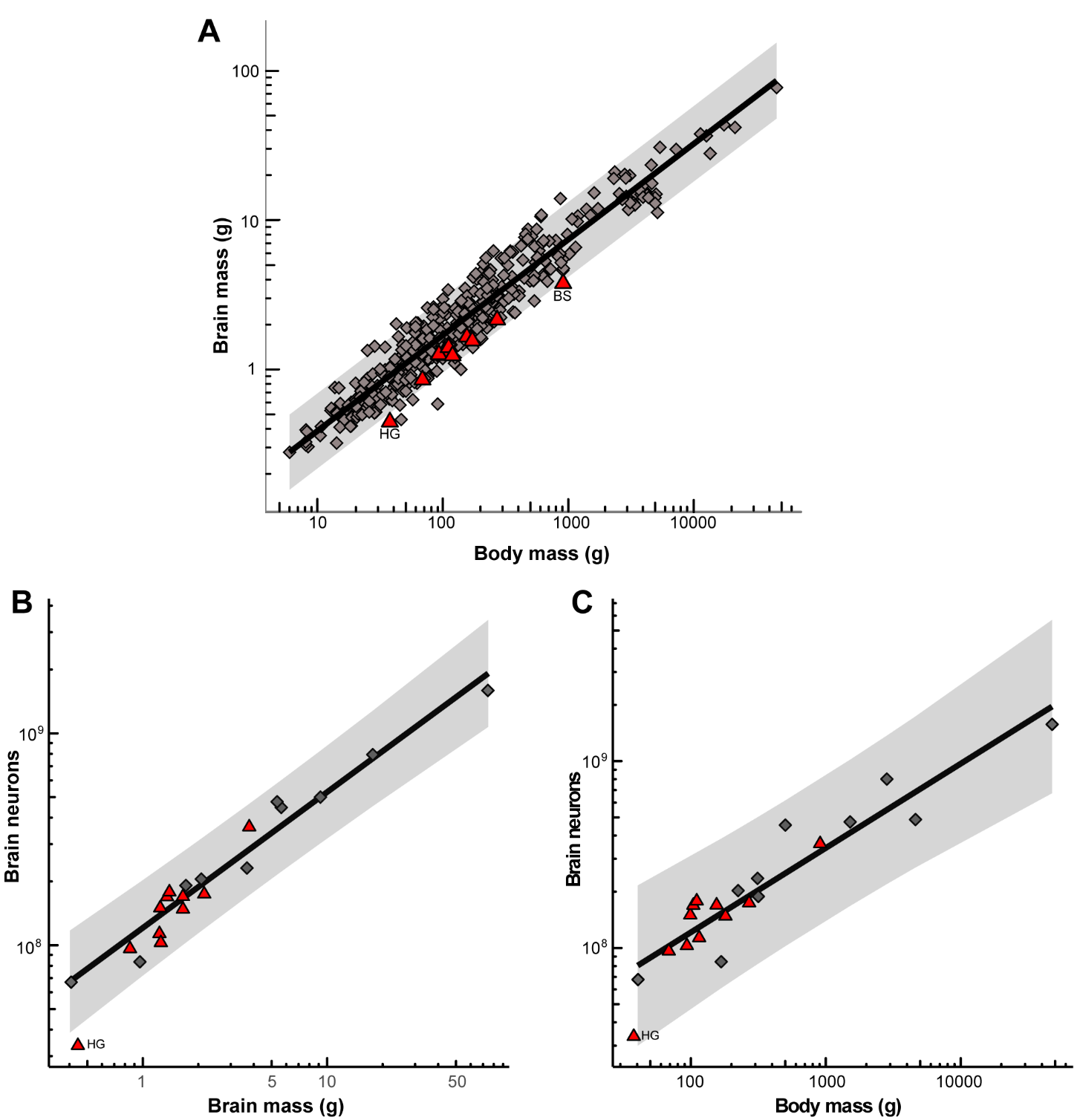
**

**Figure S1. Brain–body scaling and neuronal scaling rules for mole-rats compared with those for other rodents.** (A) Brain mass plotted as a function of body mass. (B, C) Total number of brain neurons plotted as a function of brain mass (B) and body mass (C). Each point corresponds to a species average; mole-rats are plotted as red triangles, other rodents as grey symbols. Axes are log-transformed. The fitted lines are from ordinary least squares (OLS) regressions calculated for rodents, excluding mole-rats, with the shaded areas denoting 95% prediction intervals. Data for mole-rats are from our dataset, other data on body mass and brain mass for rodents (n = 414) were collated from the literature (for references, see Dataset S1), data on number of brain neurons for other rodents are from a published report [38]. HG, *Heterocephalus glaber*.

**Table S1. Brain mass and brain region volumes**

| Species | Body mass | | Brain mass | | Olfactory bulbs | | Olfactory cortices | Neocortex | | Entorhinal cortex | | Hippocampus | | | Amygdala |  |
| --- | --- | --- | --- | --- | --- | --- | --- | --- | --- | --- | --- | --- | --- | --- | --- | --- |
| *Heterocephalus glaber* | 37.74 ± 12.02 | | 0.44 ± 0.04 | | 20 ± 3  4.92 % | | 39 ± 5  9.40 % | 100 ± 5  24.16% | | 14 ± 4  3.45 % | | 32 ± 6  7.60 % | | | 12 ± 1  2.89 % |  |
| *Cryptomys hottentotus* | 68.58 ± 12.36 | | 0.85 ± 0.06 | | 37 ± 4  4.62 % | | 50 ± 8  6.22 % | 183 ± 30  22.91 % | | 25 ± 3  3.11 % | | 59 ± 8  7.33 % | | | 14 ± 3  1.71 % |  |
| *Fukomys darlingi* | 115.19 ± 44.83 | | 1.23 ± 0.12 | | 43 ± 10  3.76 % | | 77 ± 9  6.67 % | 271 ± 19  23.46 % | | 41 ± 10  3.55 % | | 76 ± 4  6.57 % | | | 16 ± 2  1.41 % |  |
| *Cryptomys natalensis* | 99.02 ± 33.43 | | 1.24 ± 0.11 | | 55 ± 12  4.70 % | | 76 ± 12  6.55 % | 321 ± 26  27.40 % | | 43 ± 6  3.63 % | | 80 ± 13  6.79 % | | | 20 ± 4  1.67 % |  |
| *Fukomys anselli* | 93.45 ± 20.47 | | 1.25 ± 0.13 | | 55 ± 15  4.63 % | | 71 ± 9  5.97 % | 291 ± 40  24.36 % | | 38 ± 4  3.17 % | | 82 ± 23  6.82 % | | | 26 ± 5  2.16 % |  |
| *Cryptomys pretoriae* | 104.27 ± 21.31 | | 1.35 ± 0.11 | | 57 ± 9  4.46 % | | 76 ± 2  6.02 % | 315 ± 39  24.88 % | | 46 ± 4  3.60 % | | 95 ± 14  7.46 % | | | 20 ± 5  1.61 % |  |
| *Fukomys damarensis* | 110.53 ± 30.29 | | 1.39 ± 0.14 | | 59 ± 8  4.48 % | | 88 ± 8  6.73 % | 311 ± 60  23.71 % | | 44 ± 6  3.38 % | | 95 ± 12  7.24 % | | | 22 ± 2  1.69 % |  |
| *Heliophobius argent.* | 180.51 ± 35.45 | | 1.65 ± 0.12 | | 60 ± 8  3.89 % | | 107 ± 4  6.89 % | 457 ± 25  29.50 % | | 56 ± 4  3.62 % | | 115 ± 8  7.40 % | | | 34 ± 4  2.19 % |  |
| *Georychus capensis* | 155.09 ± 39.08 | | 1.65 ± 0.18 | | 74 ± 8  4.79 % | | 97 ± 13  6.23 % | 401 ± 46  25.81 % | | 53 ± 8  3.44 % | | 114 ± 19  7.32 % | | | 28 ± 3  1.83 % |  |
| *Fukomys mechowii* | 270.16 ± 52.85 | | 2.15 ± 0.24 | | 76 ± 6  3.69 % | | 133 ± 9  6.42 % | 627 ± 8  30.33 % | | 71 ± 13  3.42 % | | 137 ± 11  6.63 % | | | 35 ±10  1.69 % |  |
| *Bathyergus suillus* | 907.82± 320.93 | | 3.78 ± 0.14 | | 132 ± 14  3.70 % | | 173 ± 15  4.83 % | 1011 ± 21  28.30 % | | 96 ± 3  2.70 % | | 194 ± 1  5.44 % | | | 50 ±3  1.39 % |  |
| Species | **Striatum** | **Septum** | | **Thalamus** | | **Hypothalamus** | | | **Cerebellum** | | **Tectum** | | **Tegmentum** | **Medulla**  **oblongata** | | |
| *Heterocephalus glaber* | 29 ± 4  6.93 % | 4.1 ± 0.4  0.98 % | | 23 ± 3  5.66 % | | 17 ± 1  4.04 % | | | 48 ± 5  11.63 % | | 10 ± 3  2.32 % | | 19 ± 4  4.62 % | 47 ± 6  11.31 % | | |
| *Cryptomys hottentotus* | 59 ± 7  7.42 % | 11 ± 1  1.32 % | | 39 ± 3  4.83 % | | 25 ±6  3.12 % | | | 121 ± 19  15.06 % | | 17 ± 2  2.17 % | | 34 ± 4  4.25 % | 79 ± 2  9.87 % | | |
| *Fukomys darlingi* | 99 ± 16  8.56 % | 16 ± 3  1.41 % | | 51 ± 7  4.37 % | | 29 ± 1  2.53 % | | | 156 ± 27  13.46 % | | 25 ± 1  2.13 % | | 45 ± 5  3.94 % | 101 ± 6  8.71 % | | |
| *Cryptomys natalensis* | 84 ± 11  7.13 % | 14 ± 3  1.17 % | | 54 ± 6  4.61 % | | 31 ± 3  2.66% | | | 175 ± 19  14.87 % | | 24 ± 6  2.01 % | | 42 ± 6  3.55 % | 97 ± 18  8.28 % | | |
| *Fukomys anselli* | 84 ± 11  7.00 % | 15 ± 3  1.24 % | | 61 ± 3  5.11 % | | 35 ± 5  2.96 % | | | 200 ± 52  16.72 % | | 32 ± 6  2.66 % | | 60 ± 19  5.00 % | 132 ± 32  11.04 % | | |
| *Cryptomys pretoriae* | 85 ± 9  6.69 % | 15 ± 2  1.16 % | | 57 ± 8  4.53 % | | 33 ± 3  2.60 % | | | 188 ± 14  14.80 % | | 25 ± 4  2.01 % | | 47 ± 4  3.73 % | 119 ± 15  9.41 % | | |
| *Fukomys damarensis* | 102 ± 17  7.79 % | 15 ± 1  1.12 % | | 64 ± 7  4.88 % | | 39 ± 7  2.95 % | | | 201 ± 46  15.30 % | | 27 ± 4  2.03 % | | 56 ± 14  4.27 % | 110 ± 9  8.41 % | | |
| *Heliophobius argent.* | 127 ± 11  8.23 % | 18 ± 2  1.16 % | | 90 ± 5  5.78 % | | 42 ± 5  2.72 % | | | 215 ± 37  13.85 % | | 28 ± 3  1.83 % | | 56 ± 3  3.63 % | 120± 22  7.76 % | | |
| *Georychus capensis* | 123 ± 22  7.94 % | 21 ± 2  1.36 % | | 87 ± 16  5.58 % | | 50 ± 6  3.20 % | | | 250 ± 14  16.03 % | | 34 ± 2  2.20 % | | 62 ± 8  3.97 % | 151 ± 21  9.73 % | | |
| *Fukomys mechowii* | 198 ± 2  9.58 % | 24 ± 1  1.17 % | | 116 ± 13  5.62 % | | 60 ± 7  2.92 % | | | 391 ± 58  18.95 % | | 41 ± 1  1.97 % | | 82 ± 13  3.98 % | 242 ± 87  11.72 % | | |
| *Bathyergus suillus* | 249 ± 20  6.98 % | 35 ± 7  0.97 % | | 173 ± 15  4.83 % | | 85 ± 7  2.38 % | | | 683 ± 82  19.11 % | | 69 ± 6  1.92 % | | 156 ± 18  4.37 % | 368 ± 30  10.30 % | | |

For each species, brain mass in grams and absolute structure volumes in mm^3^ are given in the first row (values are expressed as mean ± SD) and average structure volume fractions (expressed as percentage of the whole brain volume) are given in the second row.

**Table S2. Statistical analyses of body and brain sizes and indexes of cognitive power**

| Measured variable | Differences in measured variable | | | | | |
| --- | --- | --- | --- | --- | --- | --- |
|  | **solitary vs. social, 2-level sociality** | | | **social vs. solitary, 3-level sociality** | | |
|  | **Posterior mean** | **95% CI** | **pMCMC** | **Posterior mean** | **95% CI** | **pMCMC** |
| Body mass | **1.1089** | **[0.1481, 2.2049]** | **0.0321** | -1.2621 | [-2.8323, 0.3502] | 0.0960 |
| Brain mass | 0.6486 | [-0.0018, 1.4556] | 0.0741 | -0.7759 | [-1.7588, 0.1771] | 0.1070 |
| Relative brain size | -0.02819 | [-0.1888, 1.1241] | 0.6370 | 0.0270 | [-0.1626, 0.2126] | 0.7364 |
| Neocortex ratio | 0.1376 | [-0.1234, 0.4023] | 0.2500 | -0.0850 | [-0.3523, 0.1574] | 0.4440 |
| Neuronal index | -0.2467 | [-1.7914, 1.2497] | 0.7200 | 0.0111 | [-0.6065, 0.5757] | 0.9540 |
| Cortical neurons ratio | 0.4113 | [-0.0363, 0.8235] | 0.0585 | -0.8711 | [-2.3377, 0.5066] | 0.1970 |
| Measured variable | **solitary vs. eusocial, 3-level sociality** | | | **social vs. eusocial, 3-level sociality** | | |
|  | **Posterior mean** | **95% CI** | **pMCMC** | **Posterior mean** | **95% CI** | **pMCMC** |
| Body mass | 1.0485 | [-0.4979, 2.5496] | 0.1452 | -0.1931 | [-1.2985, 0.9200] | 0.7032 |
| Brain mass | 0.6361 | [-0.3007, 1.6050] | 0.1730 | -0.1398 | [-0.8462, 0.5545] | 0.6500 |
| Relative brain size | -0.0221 | [-0.3096, 0.2150] | 0.8500 | 0.0060 | [-0.2042, 0.2158] | 0.9488 |
| Neocortex ratio | 0.1316 | [ -0.0900, 0.3716] | 0.2060 | 0.0453 | [-0.1789, 0.2934] | 0.6230 |
| Neuronal index | 0.1719 | [-0.3912, 0.7639] | 0.5116 | 0.1793 | [-0.3523, 0.7060] | 0.4524 |
| Cortical neurons ratio | 0.9896 | [-0.4165, 2.3590] | 0.1390 | 0.1352 | [-1.1479, 1.4005] | 0.8130 |

Significant results are in bold.

**Table S3. Scaling rules for brains of the African mole-rats**

| Structure |  | Scaling of structure volume with brain volume | | | |
| --- | --- | --- | --- | --- | --- |
|  | **Power law** | | **95% CI Slope** | **R^2^** | **p-value** |
| Amygdala | V_AMYG. =_ 0.0071 × V_BR._ ^0.7388^ | | [0.5133, 0.9644] | 0.859 | <0.0001 |
| Cerebellum | V_CER. =_ 0.0304 × V_BR._ ^1.2257^ | | [1.1140, 1.3374] | 0.986 | <0.0001 |
| Hippocampus | V_HIPP. =_ 0.0177 × V_BR._ ^0.8721^ | | [0.7795, 0.9646] | 0.981 | <0.0001 |
| Hypothalamus | V_HYPOT. =_ 0.1224 × V_BR._ ^0.7983^ | | [0.6649, 0.9318] | 0.953 | <0.0001 |
| Medulla oblongata | V_MO. =_ 0.0064 × V_BR._ ^0.9796^ | | [0.7879, 1.1713] | 0.937 | <0.0001 |
| Neocortex | V_NEO. =_ 0.1118 × V_BR._ ^1.1166^ | | [1.0108, 1.2225] | 0.984 | <0.0001 |
| Olfactory bulbs | V_OB._ = 0.0076 × V_BR._ ^0.8564^ | | [0.7413, 0.9713] | 0.969 | <0.0001 |
| Olfactory cortices | V_OB. =_ 0.1006 × V_BR._ ^0.7568^ | | [0.6338, 0.8797] | 0.956 | <0.0001 |
| Septum | V_SEP. =_ 0.00005 × V_BR._ ^0.9825^ | | [0.8141, 1.1510] | 0.951 | <0.0001 |
| Entorhinal cortex | V_ENT. =_ 0.0012 × V_BR._ ^0.9300^ | | [0.8170, 1.0431] | 0.975 | <0.0001 |
| Striatum | V_STR. =_ 0.0010 × V_BR._ ^1.0570^ | | [0.9089, 1.2050] | 0.967 | <0.0001 |
| Tectum | V_TEC. =_ 0.0007 × V_BR._ ^0.9018^ | | [0.7778, 1.0258] | 0.968 | <0.0001 |
| Tegmentum | V_TEG. =_ 0.0013× V_BR._ ^0.9564^ | | [0.8133, 1.0995] | 0.962 | <0.0001 |
| Thalamus | V_THAL. =_ 0.0013 × V_BR._ ^0.9882^ | | [0.8485, 1.1280] | 0.966 | <0.0001 |
| Brain part | **Scaling of neuronal numbers with structure mass** | | | | |
|  | **Power law** | | **95% CI Slope** | **R^2^** | **p-value** |
| Whole brain | N_BR =_ 74,730 × M_BR._ ^1.0389^ | | [0.7779, 1.3000] | 0.900 | <0.0001 |
| Cortex | N_CX. =_ 30,577 × M_CX_ ^0.9980^ | | [0.7134, 1.2827] | 0.875 | <0.0001 |
| Cerebellum | N_CER. =_ 486,284 × M_CER._ ^0.9854^ | | [0.8577, 1.1130] | 0.971 | <0.0001 |
| Subcortical forebrain | N_SFB =_ 43,082 × M_SFB._ ^0.9157^ | | [0.5874, 1.2439] | 0.816 | <0.0001 |
| Olfactory bulbs | N_OB. =_ 216,575 × M_OB._ ^0.9344^ | | [0.7604, 1.1084] | 0.943 | <0.0001 |
| Brain stem | N_BS =_ 113,632 × M_BS._ ^0.6805^ | | [0.4514, 0.9095] | 0.834 | <0.0001 |
| Brain part | **Scaling of nonneuronal cell numbers with structure mass** | | | | |
|  | **Power law** | | **95% CI Slope** | **R^2^** | **p-value** |
| Whole brain | OC_BR =_ 119,174 × M_BR._ ^0.9246^ | | [0.8284, 1.0208] | 0.981 | <0.0001 |
| Cortex | OC_CX. =_ 37,413 × M_CX_ ^1.0195^ | | [0.8491, 1.1899] | 0.953 | <0.0001 |
| Cerebellum | OC_CER. =_ 403,487 × M_CER._ ^0.7673^ | | [0.6518, 0.8828] | 0.961 | <0.0001 |
| Subcortical forebrain | OC_SFB =_ 58,357 × M_SFB._ ^1.0131^ | | [0.8096, 1.2166] | 0.934 | <0.0001 |
| Olfactory bulbs | OC_OB. =_ 180,813 × M_OB._ ^0.9771^ | | [0.8771, 1.0771] | 0.982 | <0.0001 |
| Brain stem | OC_BS =_ 207,629 × M_BS._ ^0.8077^ | | [0.6083, 1.0071] | 0.903 | <0.0001 |

**Table S4. Statistical analyses of brain region volumes**

| Structure | Differences in relative structure volume | | | | | |
| --- | --- | --- | --- | --- | --- | --- |
|  | **solitary vs. social, 2-level sociality** | | | **social vs. solitary, 3-level sociality** | | |
|  | **Posterior mean** | **95% CI** | **pMCMC** | **Posterior mean** | **95% CI** | **pMCMC** |
| Amygdala | 0.1458 | [-0.1217, 0.3849] | 0.216 | -0.2094 | [-0.5256, 0.0794] | 0.131 |
| Cerebellum | -0.0749 | [-0.2200, 0.0651] | 0.264 | 0.1050 | [-0.0790, 0.2954] | 0.235 |
| Entorhinal cortex | -0.0188 | [-0.2273, 0.2042] | 0.849 | 0.0548 | [-0.2254, 0.3288] | 0.638 |
| Hippocampus | 0.0012 | [-0.1740, 0.1670] | 1.000 | 0.0367 | [-0.1782, 0.2445] | 0.696 |
| Hypothalamus | 0.0571 | [-0.0837, 0.2037] | 0.381 | -0.0987 | [-0.2857, 0.0674] | 0.232 |
| Medulla oblongata | -0.0459 | [-0.2844, 0.2000] | 0.654 | 0.0110 | [-0.2973, 0.3173] | 0.939 |
| Neocortex | 0.0197 | [-0.1089, 0.1522] | 0.740 | 0.0046 | [-0.1594, 0.1737] | 0.952 |
| Olfactory bulbs | 0.0047 | [-0.1711, 0.1961] | 0.974 | 0.0542 | [-0.1667, 0.2717] | 0.570 |
| Olfactory cortices | 0.0292 | [-0.1254, 0.1977] | 0.692 | -0.0870 | [-0.2754, 0.1167] | 0.325 |
| Septum | -0.0413 | [-0.3293, 0.2385] | 0.746 | 0.0824 | [-0.3124, 0.4495] | 0.620 |
| Striatum | 0.0110 | [-0.2171, 0.1686] | 0.910 | -0.0241 | [-0.2840, 0.2386] | 0.820 |
| Tectum | -0.0218 | [-0.2534, 0.2195] | 0.845 | 0.0108 | [-0.2873, 0.2985] | 0.932 |
| Tegmentum | -0.0129 | [-0.1986, 0.1858] | 0.893 | -0.0260 | [-0.2595, 0.2262] | 0.787 |
| Thalamus | 0.0799 | [-0.0369, 0.2099] | 0.174 | -0.1067 | [-0.2612, 0.0501] | 0.148 |
| Structure | **solitary vs. eusocial, 3-level sociality** | | | **social vs. eusocial, 3-level sociality** | | |
|  | **Posterior mean** | **95% CI** | **pMCMC** | **Posterior mean** | **95% CI** | **pMCMC** |
| Amygdala | 0.0996 | [-0.1745, 0.3830] | 0.432 | -0.1197 | [-0.3964, 0.1568] | 0.327 |
| Cerebellum | -0.0539 | [-0.2184, 0.1220] | 0.467 | 0.0526 | [-0.1130, 0.2224] | 0.482 |
| Entorhinal cortex | 0.0066 | [-0.2440, 0.2560] | 0.956 | 0.0682 | [-0.1770, 0.3219] | 0.544 |
| Hippocampus | 0.0263 | [-0.1631, 0.2239] | 0.759 | 0.0630 | [-0.1376, 0.2517] | 0.456 |
| Hypothalamus | 0.0300 | [-0.1311, 0.1855] | 0.675 | -0.0690 | [-0.2350, 0.0900] | 0.334 |
| Medulla oblongata | -0.0592 | [-0.3345, 0.2405] | 0.608 | -0.0505 | [-0.3222, 0.2594] | 0.657 |
| Neocortex | 0.0348 | [ -0.1232, 0.1892] | 0.602 | 0.0392 | [-0.1244, 0.1924] | 0.555 |
| Olfactory bulbs | 0.0457 | [-0.1648, 0.2519] | 0.626 | 0.0988 | [-0.1115, 0.3100] | 0.280 |
| Olfactory cortices | -0.0057 | [-0.1829, 0.1841] | 0.953 | -0.0877 | [-0.2809, 0.0912] | 0.286 |
| Septum | -0.0140 | [-0.3777, 0.3554] | 0.944 | 0.0693 | [-0.3060, 0.3920] | 0.645 |
| Striatum | -0.0356 | [-0.2643, 0.2085] | 0.732 | -0.0600 | [-0.3057, 0.1701] | 0.583 |
| Tectum | -0.0309 | [-0.3046, 0.2448] | 0.806 | -0.0149 | [-0.2893, 0.2555] | 0.894 |
| Tegmentum | -0.0422 | [-0.2456, 0.1833] | 0.640 | -0.0713 | [-0.2767, 0.1445] | 0.444 |
| Thalamus | 0.0576 | [-0.0883, 0.1947] | 0.368 | -0.0501 | [-0.1936, 0.0844] | 0.436 |

Structures are listed alphabetically.

**Table S5. Numbers of neurons in the whole brain and the major brain divisions**

| Species | Whole brain,  × 10^6^ | Olfactory bulbs, × 10^6^ | Cortex,  × 10^6^ | Subcortical forebrain, × 10^6^ | Cerebellum,  × 10^6^ | Brain stem,  × 10^6^ |
| --- | --- | --- | --- | --- | --- | --- |
| *Heterocephalus glaber* | 33.62 ± 3.66 | 3.01 ± 0.33 | 5.31 ± 0.31 | 2.86 ± 0.50 | 20.52 ± 3.01 | 1.91 ± 0.28 |
| *Cryptomys hottentotus* | 96.24 ± 9.65 | 7.99 ± 0.46 | 11.73 ± 0.22 | 4.55 ± 0.69 | 68.81 ± 9.02 | 3.17 ± 0.36 |
| *Fukomys darlingi* | 113.35 ± 3.79 | 7.60 ± 1.25 | 12.49 ± 0.53 | 5.19 ± 0.81 | 84.91± 3.95 | 3.14 ± 0.15 |
| *Cryptomys natalensis* | 149.98 ± 8.50 | 11.85 ± 0.29 | 17.46 ± 1.39 | 6.82 ± 0.81 | 109.75 ± 8.75 | 4.11 ± 0.14 |
| *Fukomys anselli* | 102.82 ± 12.28 | 8.88 ± 0.64 | 9.73 ± 2.23 | 4.86 ± 0.90 | 75.44 ± 8.90 | 3.90 ± 1.16 |
| *Cryptomys pretoriae* | 169.16 ± 16.23 | 12.07 ± 1.41 | 20.56 ± 0.90 | 9.92 ± 0.62 | 120.58 ± 16.69 | 6.03 ± 0.35 |
| *Fukomys damarensis* | 178.3 ± 19.08 | 12.55 ± 1.82 | 21.27 ± 0.59 | 9.58 ± 1.69 | 129.96 ± 18.15 | 4.94 ± 1.10 |
| *Heliophobius argent.* | 147.94 ± 6.80 | 10.30 ± 2.15 | 25.44 ± 2.23 | 11.92 ± 1.13 | 94.82 ± 1.97 | 5.46 ± 0.84 |
| *Georychus capensis* | 169.67 ± 11.42 | 12.46 ± 1.42 | 25.60 ± 2.09 | 9.06 ± 1.13 | 116.74 ± 10.31 | 5.81 ± 0.34 |
| *Fukomys mechowii* | 174.24 ± 14.60 | 14.11 ± 2.13 | 23.32 ± 0.94 | 9.42 ± 0.88 | 122.64 ± 14.34 | 4.75 ± 0.98 |
| *Bathyergus suillus* | 361.29 ± 24.4 | 23.81 ± 2.96 | 43.48 ± 5.11 | 16.42 ± 0.75 | 268.89 ± 17.47 | 8.67 ± 0.60 |

Species ordered by increasing brain size. All values are given as mean ± SD.

**Table S6. Statistical analyses of neuronal numbers**

| Brain part | Differences in the absolute number of neurons | | | Differences in the relative number of neurons | | |
| --- | --- | --- | --- | --- | --- | --- |
|  | **solitary vs. social, 2-level sociality** | | | **solitary vs. social, 2-level sociality** | | |
|  | **Posterior mean** | **95 % CI** | **pMCMC** | **Posterior mean** | **95 % CI** | **pMCMC** |
| Whole brain | 0.5982 | [-0.3312, 1.4613] | 0.1630 | -0.0534 | [-0.5521, 0.4563] | 0.7828 |
| Cortex | **0.7928** | **[0.0694, 1.5191]** | **0.0396** | 0.2052 | [-0.3593, 0.7809] | 0.3919 |
| Cerebellum | 0.5692 | [-0.4279, 1.5111] | 0.2130 | -0.1157 | [-0.7093, 0.4330] | 0.6241 |
| Subcortical forebrain | **0.6884** | **[0.0306, 1.3882]** | **0.0480** | 0.1538 | [-0.4345, 0.7264] | 0.5233 |
| Olfactory bulbs | 0.4878 | [-0.3242, 1.2337] | 0.1940 | -0.1103 | [-0.5395, 0.3418] | 0.5635 |
| Brain stem | 0.5463 | [-0.0172, 1.0851] | 0.0484 | 0.1382 | [-0.2760, 0.6170] | 0.4649 |
| Brain part | **social vs. solitary, 3-level sociality** | | | **social vs. solitary, 3-level sociality** | | |
|  | **Posterior mean** | **95 % CI** | **pMCMC** | **Posterior mean** | **95 % CI** | **pMCMC** |
| Whole brain | -0.4842 | [-0.4619, 1.5675] | 0.3000 | 0.1030 | [-0.2653, 0.4744] | 0.508 |
| Cortex | -0.6908 | [-1.5711, 0.1442] | 0.1060 | -0.2966 | [-0.8320, 0.2030] | 0.2140 |
| Cerebellum | -0.4463 | [-0.5476, 0.6607] | 0.3950 | 0.2400 | [-0.1244, 0.6178] | 0.1790 |
| Subcortical forebrain | -0.6415 | [-1.5070, 0.1206] | 0.1076 | -0.2420 | [-0.7458, 0.2780] | 0.3068 |
| Olfactory bulbs | -0.4013 | [-1.3135, 0.5283] | 0.3260 | 0.1669 | [-0.2029, 0.5381] | 0.3380 |
| Brain stem | -0.4945 | [-1.1205, 0.1411] | 0.1028 | -0.1214 | [-0.4713, 0.2597] | 0.4792 |
| Brain part | **solitary vs. eusocial, 3-level sociality** | | | **solitary vs. eusocial, 3-level sociality** | | |
|  | **Posterior mean** | **95 % CI** | **pMCMC** | **Posterior mean** | **95 % CI** | **pMCMC** |
| Whole brain | 0.7093 | [-0.3204, 1.7492] | 0.1550 | -0.0175 | [-0.3873, 0.3655] | 0.8730 |
| Cortex | 0.8823 | [-0.0597, 1.7186] | 0.0480 | 0.3752 | [-0.1891, 0.9542] | 0.1480 |
| Cerebellum | 0.7095 | [-0.3922, 1.7841] | 0.1840 | -0.1225 | [-0.4990, 0.2778] | 0.4590 |
| Subcortical forebrain | 0.7157 | [-0.1268, 1.4780] | 0.0736 | 0.1981 | [-0.3445, 0.7246] | 0.4276 |
| Olfactory bulbs | 0.5580 | [-0.3500, 1.4611] | 0.1980 | -0.1623 | [-0.5780, 0.2267] | 0.3804 |
| Brain stem | **0.5932** | **[0.0070, 1.2574]** | **0.0584** | 0.1255 | [-0.2617, 0.4965] | 0.4576 |
| Brain part | **social vs. eusocial, 3-level sociality** | | | **social vs. eusocial, 3-level sociality** | | |
|  | **Posterior mean** | **95 % CI** | **pMCMC** | **Posterior mean** | **95 % CI** | **pMCMC** |
| Whole brain | 0.2357 | [-0.7411, 1.1657] | 0.5880 | 0.0893 | [-0.2077, 0.3977] | 0.5030 |
| Cortex | 0.1885 | [-0.6152, 1.0791] | 0.6214 | 0.0771 | [-0.3592, 0.5090] | 0.6968 |
| Cerebellum | 0.2937 | [-0.7110, 1.3721] | 0.5360 | 0.1133 | [-0.1955, 0.4269] | 0.4080 |
| Subcortical forebrain | 0.0574 | [-0.7410, 0.7963] | 0.8456 | -0.0450 | [-0.4409, 0.3785] | 0.8100 |
| Olfactory bulbs | 0.1623 | [-0.7803, 1.0480] | 0.6680 | 0.0067 | [-0.3226, 0.3078] | 0.9604 |
| Brain stem | 0.0998 | [-0.4819, 0.7439] | 0.7175 | 0.0041 | [-0.3167, 0.2861] | 0.9704 |

Significant results are in bold.

**Table S7. Mean and maximum group size for mole-rat species**

| Species | Mean group size | Max group size | References |
| --- | --- | --- | --- |
| *Heterocephalus glaber* | 60 | 295 | Brett 1991 |
| *Cryptomys hottentotus* | 8 | 16 | Bennett 1989 |
| *Fukomys darlingi* | 5 | 9 | Bennett et al. 1994 |
| *Cryptomys natalensis* | 8 | 9 | Jarvis and Bennett 1991, Oosthuizen et al. 2008 |
| *Fukomys anselli* | 10 | 16 | Šklíba et al. 2012, Sichilima et al 2011 |
| *Cryptomys pretoriae* | 10 | 12 | Moolman et al. 1998 |
| *Fukomys damarensis* | 12 | 41 | Jarvis and Bennett, 1993, |
| *Heliophobius argent.* | 1 | 1 | Bennett and Faulkes 2000, Šumbera et al. 2003 |
| *Georychus capensis* | 1 | 1 | Bennett and Jarvis 1988 |
| *Fukomys mechowii* | 10 | 20 | Šumbera et al. 2012, Sichilima et al 2008, Scharff et al 2001 |
| *Bathyergus suillus* | 1 | 1 | Bennett and Faulkes 2000, Hart et al. 2006 |

**References:**

Bennett, N. C. The social structure and reproductive biology of the common mole-rat, *Cryptomys h. hottentotus* and remarks on the trends in reproduction and sociality in the family Bathyergidae. *J. Zool.* **219**, 45-59. (1989).

Bennett, N. C. & Faulkes, C. G. African mole-rats: ecology and eusociality. (Cambridge University Press, 2000).

Bennett, N. C. & Jarvis, J. U. M. The reproductive biology of the Cape mole-rat, *Georychus capensis* (Rodentia, Bathyergidae). *J. Zool.* **214**, 95-106 (1988).

Bennett, N. C., Jarvis, J.U.M. & Cotterill, F. P. D. The colony structure and reproductive biology of the Mashona mole-rat, *Cryptomys darlingi* from Zimbabwe. *J. Zool.* **234**, 477-487 (1994).

Brett, R. A. The population structure of naked mole-rat colonies in *The biology of the baked mole-rat* (eds. Sherman, P. W., Jarvis, J. U. M., Alexander, R. D.) 97-136 (Princeton University Press, 1991).

Hart, L., O'Riain, M. J., Jarvis, J. U. M. & Bennett N. C. Is the Cape dune mole-rat, *Bathyergus suillus* a seasonal or aseasonal breeder? *J. Mammal.* **87**, 1078-1085 (2006).

Jarvis, J. U. M. & Bennett, N. C. Ecology and behavior of the family Barthyergidae in *The biology of the baked mole-rat* (eds. Sherman, P. W., Jarvis, J. U. M., Alexander, R. D.) 66-96 (Princeton University Press, 1991).

Jarvis, J. U. M. & Bennett, N. C. Eusociality has evolved independently in two genera of bathyergid mole-rats—but occurs in no other subterranean mammal. *Behav. Ecol. Sociobiol.* **33**, 253-260 (1993).

Moolman, M., Bennett, N. C. & Schoeman, A. S. The social structure and dominance hierarchy of the Highveld mole-rat, *Cryptomys hottentotus pretoriae*, (Rodentia: Bathyergidae). *J. Zool.* **246**, 193-201 (1998).

Oosthuizen, M. K., Lutermann, H., Coen, C. W. & Bennett, N.C. Reproductive suppression in subordinate Natal mole-rats (*Cryptomys hottentotus natalensis*): the pituitary response to exogenous GnRH. *Gen. Comp. Endocrinol.* **159**, 236-240 (2008).

Scharff, A., Locker-Grutjen, O., Kawalika, M. & Burda, H. Natural history of the giant mole-rat, *Cryptomys mechowi* (Rodentia: Bathyergidae), from Zambia. *J. Mammal.* **82**, 1003–1015 (2001).

Sichilima, A. M., Bennett, N. C., Faulkes, C. G. & Le Comber, S. C. Evolution of African mole-rat sociality: burrow architecture, rainfall and foraging in colonies of the cooperatively breeding *Fukomys mechowii*. *J. Zool.* **275**, 276-282 (2008).

Sichilima, A. M., Bennett, N. C. & Faulkes, C. G. Field evidence for colony size and aseasonality of breeding and in Ansell’s mole-rat, *Fukomys anselli* (Rodentia: Bathyergidae). *Afr. Zool.* **46**, 334-339 (2011).

Šklíba, J., Mazoch, V., Patzenhauerová, H., Hrouzková, E., Lövy, M., Kott, O. & Šumbera, R. A maze-lover’s dream: Burrow architecture, natural history and habitat characteristics of Ansell’s mole-rat (*Fukomys anselli*). *Mamm. Biol.* **77**, 420-427 (2012).

Šumbera, R., Burda, H. & Chitaukali, W. N. Reproductive biology of a solitary subterranean bathyergid rodent, the silvery mole-rat, *Heliophobius argenteocinereus. J. Mammal.* **84**, 278-287 (2003).

Šumbera, R., Mazoch, V., Patzenhauerová, H., Lövy, M., Šklíba, J., Bryja, J. & Burda, H. Burrow architecture, family composition and habitat characteristics of the largest social African mole-rat; the giant mole-rat constructs really giant burrow systems. *Acta Theriol.* **57**, 121-130 (2012).
